# Broad time‐dependent transcriptional activity of metabolic genes of *E. coli* O104:H4 strain C227/11Φcu in a soil microenvironment at low temperature

**DOI:** 10.1111/1758-2229.13198

**Published:** 2023-08-29

**Authors:** Katharina Detert, Jonathan Währer, Kay Nieselt, Herbert Schmidt

**Affiliations:** ^1^ Department of Food Microbiology and Hygiene, Institute of Food Science and Biotechnology University of Hohenheim Stuttgart Germany; ^2^ Institute for Bioinformatics and Medical Informatics University of Tübingen Tübingen Germany

## Abstract

In the current study, metabolic genes and networks that influence the persistence of pathogenic *Escherichia coli* O104:H4 strain C227/11Φcu in agricultural soil microenvironments at low temperature were investigated. The strain was incubated in alluvial loam (AL) and total RNA was prepared from samples at time point 0, and after 1 and 4 weeks. Differential transcriptomic analysis was performed by RNA sequencing analysis and values obtained at weeks 1 and 4 were compared to those of time point 0. We found differential expression of more than 1500 genes for either time point comparison. The two lists of differentially expressed genes were then subjected to gene set enrichment of Gene Ontology terms. In total, 17 GO gene sets and 3 Pfam domains were found to be enriched after 1 week. After 4 weeks, 17 GO gene sets and 7 Pfam domains were statistically enriched. Especially stress response genes and genes of the primary metabolism were particularly affected at both time points. Genes and gene sets for uptake of carbohydrates, amino acids were strongly upregulated, indicating adjustment to a low nutrient environment. The results of this transcriptome analysis show that persistence of C227/11Φcu in soils is associated with a complex interplay of metabolic networks.

## Graphical abstract

RNAseq analysis was used to study the changes in the differential transcriptomic profile of *Escherichia coli* O104:H4 strain C227/11Φcu in agricultural soil microenvironments at time point 0, and after 1 and 4 weeks at low temperature. Especially genes associated with stress response and primary metabolism genes were particularly affected at time points 1 and 4 weeks, as well as genes and gene sets for uptake of carbohydrates and amino acids, indicating adjustment to a low nutrient environment.

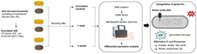

## INTRODUCTION

During recent years, outbreaks caused by enterohemorrhagic *Escherichia coli* (EHEC), and hybrid enterohemorrhagic/enteroaggregative *E. coli* (EHEC/EAEC) were increasingly correlated with the consumption of non‐heated vegetables (Grant et al., 2008; Greig & Ravel, 2009; Hilborn et al., 1999; Marder et al., 2014; Waltenburg et al., 2022). In 2011, a novel hybrid EHEC/EAEC strain of serotype O104:H4 caused a large outbreak of disease in Germany and fenugreek sprouts were identified as the most probable source of infection (King et al., 2012; Robert Koch Institute, 2011). EHEC strains produce at least one phage‐encoded Shiga toxin and can cause diarrhoea and serious sequelae such as hemorrhagic colitis and the hemolytic‐uremic syndrome (HUS) (Boyce et al., 1995; Currie et al., 2007; King et al., 2012; Nataro & Kaper, 1998).

Shiga‐toxin producing *E. coli* strains (STEC) are ubiquitous distributed on farms since cattle harbour these pathogens in their gastrointestinal tract and are regarded as the primary reservoir (Beuchat, 2002; Chapman et al., 1993; Persad & LeJeune, 2014; Reddy et al., 1981). Consequently, the use of cattle manure for soil fertilization is an important route for crop plant contamination with pathogenic *E. coli* (McCabe et al., 2019; Renter et al., 2003). The survival of faecal pathogens in agricultural soil has been investigated in several studies, which showed that EHEC strains could persist for several weeks (Baker et al., 2020, 2021; Detert & Schmidt, 2021; Islam et al., 2004; Underthun et al., 2018; Zhang et al., 2013). Thereby, the dependence on biotic and abiotic factors was mainly investigated. In a previous study, we showed that the survival of *E. coli* O104:H4 strain C227/11Φcu dependeds on soil type, temperature and nutrient availability (Detert & Schmidt, 2021). In addition, genetic factors that might influence the survival in soil microenvironments were investigated by using isogenic deletion mutants of C227/11Φcu. Thereby, the sigma factor RpoS, which is the master regulator of the general stress response was identified as an important determinant for soil survival. To better understand, how *E. coli* adapts to conditions in the soil microenvironment, Duffitt et al. (2011) compared genetic expression profiles of *Escherichia coli* O157:H7 in sterile soil at 15°C to expression in LB medium using DNA microarrays. The authors found an enhanced expression of genes for e.g., stress response, antibiotic resistance, biosynthesis, metabolism, virulence and ribosomal proteins after 2 weeks of incubation. Tao et al. (1999) compared the gene expression of *E. coli* K12 in minimal medium to LB medium. In response to nutrient limitation, genes correlated with stress response, acetate metabolism and amino acid biosynthesis were highly expressed.

The aim of the current study was to analyse the complex metabolic networks that are necessary for adaptation and persistence of *E. coli* O104:H4 strain C227/11Φcu (Zangari et al., 2013) in soil in defined agricultural soil microenvironments in a laboratory scale. We used autoclaved alluvial loam (AL) and incubated the samples at 4°C, since the longest survival was found at these conditions in our earlier study (Detert & Schmidt, 2021). In order to perform differential transcriptomic analysis, total RNA was isolated from soil samples immediately after inoculation, and after 1 and 4 weeks post inoculation.

## MATERIALS AND METHODS

### Bacterial strain and growth media

All *E. coli* strains used in this study are shown in Table 1. They were routinely cultivated in LB medium (pH 7.0) consisting of 10 g/L tryptone, 10 g/L NaCl and 5 g/L yeast extract, and aerated by vigorous shaking on a rotary shaker at 37°C with 180 rpm. When required, the medium was adjusted to a final concentration of 50 μg/mL kanamycin (kanamycin sulfate, Roth) or 25 μg/mL chloramphenicol (Roth). For preparation of LB agar plates, 15 g/L agar was added to the liquid medium prior to autoclaving at 121°C for 15 min. TBX agar (Roth) was prepared according to the manufacturers recommendation and autoclaved as described above.

**TABLE 1:** *Escherichia coli* strains and plasmids used in this study.

Strain or plasmid	Characteristics	Origin
**Strains**		
*E. coli* O104:H4 strain C227/11Φcu	*stx*_2a_‐phage cured outbreak strain from 2011	(Zangari et al., 2013)
*E. coli* O104:H4 strain C227/11Φcu Δ*acs*	Deletion of *acs*	This study
**Plasmids**		
pKEC1.5	Derivative of plasmid pKD46, ampicillin resistance replaced by chloramphenicol resistance	(Saile et al., 2018)
pKD4	Carries kanamycin resistance cassette flanked by FRT sites	(Datsenko & Wanner, 2000)
pCP20	Encoding for FLP recombinase, temperature‐sensitive, ampicillin and chloramphenicol resistance	(Datsenko & Wanner, 2000)

#### Construction of a gene deletion mutant

The lambda red recombinase system was used to prepare an isogenic deletion mutant strain of C227/11Φcu as described in earlier studies (Datsenko & Wanner, 2000; Saile et al., 2018). Since C227/11Φcu is resistant to ampicillin, the helper plasmid pKEC1.5 (Table 1) was used instead of pKD46 (Saile et al., 2018). Mutagenesis of the gene *acs* was performed using the plasmids and primers shown in Tables 1 and 2, respectively. Successful gene deletion was confirmed by PCR using the primer listed in Table 2.

**TABLE 2:** Oligonucleotide primers used for the construction of *acs* gene deletion mutant.

Name	Sequence (5′–3′)^a^	Function
*acs*del‐O104‐for	**ATGAGCCAAATTCACAAACACACCATTCCTGCCAACATCG**gcgattgtgtaggctggagc	Mutagenesis
*acs*del‐O104‐rev	**TTACGATGGCATCGCGATAGCCTGCTTCTCTTCAAGCAGC**catggtccatatgaatatcctcc	Mutagenesis
det‐*acs*‐O104‐for	GTGCGTTTATTTTTATCCTTGTCAT	Confirmation of mutagenesis
det‐*acs*‐O104‐rev	CGCTGATAAATAGTGCCATTCAT	Confirmation of mutagenesis

^a^ The homologous regions for recombineering are highlighted in bold.

#### Soil microenvironment inoculation experiments

The survival of C227/11Φcu and the corresponding Δ*acs* mutant was investigated in soil microenvironments as described earlier with minor modifications (Detert & Schmidt, 2021). Briefly, alluvial loam (AL), kindly provided by Dr. Rita Grosch (Leibniz Institute of Vegetable and Ornamental Crops, Großbeeren, Germany) was used for the experiments. AL is described as gleyic‐fluvisol with heavy sandy loam and 27.5% clay (Rühlmann & Ruppel, 2005; Schreiter et al., 2014) and was already used in former studies (Detert & Schmidt, 2021; Eißenberger et al., 2018; Jechalke et al., 2019; Schreiter et al., 2014). AL samples of 25 g were autoclaved at 121°C for 15 min, and inoculated afterwards with 10^8^ colony forming units/g soil dry weight (cfu/g soil). As controls, untreated AL samples were used for inoculation. The inoculated soil samples were incubated at 4°C and 22°C for up to 12 weeks. To determine the cfu/g soil, the samples were plated directly after inoculation (day 0) and after 1, 4 and 12 weeks on TBX agar as described previously (Detert & Schmidt, 2021). Each treatment was repeated with two biological replicates.

#### RNA isolation from inoculated soil for transcriptomic analysis

To determine the transcriptional activity of *E. coli* O104:H4 C227/11Φcu in AL during long‐term persistence, total RNA was isolated from inoculated soil samples. The inoculation of autoclaved AL samples was carried out as described above with minor modifications. Here, AL samples were inoculated with 10^9^ cfu/g soil dry weight to ensure sufficient RNA concentrations required for RNA‐seq. The survival of C227/11Φcu was again verified by determining the cfu/g soil directly after inoculation immediately after inoculation at time point 0, and after 1 and 4 weeks. At these time points, RNA was isolated from the autoclaved and inoculated soil samples. As controls for the survival study, untreated AL samples were inoculated with 10^8^ cfu/g dw as described above. These samples were not further used for RNA isolation. RNA was isolated with the RNeasy PowerSoil Total RNA Kit (Qiagen) according to the manufacturer's recommendations (RNeasy PowerSoil Total RNA Kit Handbook 06/2017). Afterwards, the eluted RNA was directly treated with a DNA‐free kit (Thermo Fisher Scientific, USA) according to the user guide (Life Technologies Corporation, October 2012) to guarantee complete digestion of genomic DNA. Quality and quantity of total RNA were determined by agarose gel electrophoresis and the RNA samples were stored at −70°C until further procedure.

### Differential transcriptomic analysis

A total amount of 400 ng RNA was subjected to rRNA depletion for each replicate. The Illumina™ Stranded Total RNA Prep Ligation with Ribo Zero Plus Kit for ribosomal RNA depletion and cDNA library construction was used according to the manufacturer's instructions. Libraries were sequenced as single‐reads (101 bp read length) on a NovaSeq 6000 platform (Illumina) at a depth of 10.1–24.9 million reads per sample. Library preparation and sequencing procedures were performed by the same individual and a design aimed to minimize technical batch effects was chosen. RNA sequencing was performed by the Institute for Medical Microbiology (part of the NGS Competence Center NCCT, Tübingen, Germany) while data management including data storage of raw data for this project were carried out by the Quantitative Biology Center (QBiC, Tübingen, Germany).

Sequencing statistics including the quality per base and adapter content assessment of resulting transcriptome sequencing data were conducted with FastQC v0.11.5 (Andrews, 2010). All reads mappings were performed against the reference sequence of *E. coli* O104:H4 strain C227/11 isolate 368 (RefSeq ID NZ_CP011331.1) including its unknown plasmids (NZ_CP011332.1 to NZ_CP011338.1). The mappings of all samples were conducted with HISAT2 v2.1.0 (Kim et al., 2015). Spliced alignment of reads was disabled and library type was set to reverse (HISAT2 parameter—no‐spliced‐alignment and—rna‐strandness R). The resulting mapping files in SAM format were converted to BAM format using SAMtools v1.9 (Li et al., 2009). Mapping statistics, including percentage of mapped reads and fraction exonic region coverage, were conducted with the RNA‐Seq module of QualiMap2 v2.2.2‐dev (Okonechnikov et al., 2016). The total number and percentage of reads for all sequencing reactions mapped to *E. coli* are depicted in Table S1. Gene counts for all samples were computed with featureCounts v1.6.4 (Liao et al., 2014) based on the annotation of the respective reference genome, where the selected feature type was set to transcript records (featureCounts parameter ‐t transcript). To assess variability of the replicates of each condition, a principal component analysis (PCA) was conducted with the DESeq2 package v1.20.0 (Love et al., 2014).

For the computation of genes differentially expressed between the samples immediately after inoculation (time point 0), week 1 and week 4, DESeq2 v1.20.0 (Love et al., 2014) was applied to the absolute gene counts as computed with featureCounts. DESeq computes differentially expressed genes based on a model using the negative binomial distribution and internally normalizes for library depth. Genes with low counts (less than 10 reads) over all replicates were filtered prior to differential expression analysis. For differences between each condition genes with an adjusted *p*‐value (FDR) < 0.05 and absolute log_2_ fold change (FC) ≥ 1 were reported as differentially expressed. Enrichment analysis was carried out with locus tags and log_2_FC of differentially expressed genes using FUNAGE‐Pro (https://doi.org/10.1093/nar/gkac441) and *E. coli* O104:H4 strain C227/11 as reference organism.

All RNA‐seq Illumina read files as well as the unnormalized counts have been deposited in NCBI's Gene Expression Omnibus (GEO) and are available under the accession number GSE222589.

## RESULTS

### *Persistence of* E. coli *O104:H4 C227/11Φcu in autoclaved soil microenvironments*

**FIGURE 1:**
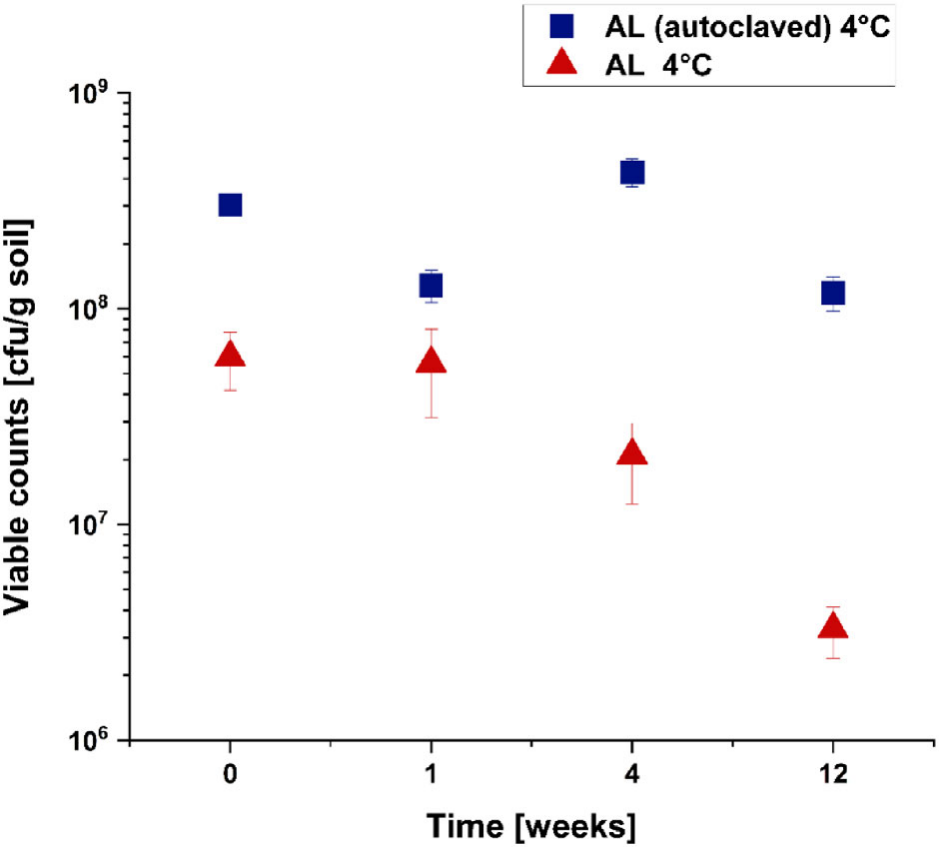
Survival of *Escherichia coli* O104:H4 strain C227/11Φcu in soil microenvironments consisting of 25 g autoclaved AL and non‐autoclaved AL soil at 4°C (as indicated). The soil was inoculated with 10^9^ (autoclaved) or 10^8^ (non‐autoclaved) cfu/g soil and incubated for 12 weeks. The experiment was performed in two biological duplicates. Error bars show the standard deviation of a biological replicate.

Since strain C227/11Φcu has been used as a model strain in recent plant and soil survival studies (Bufe et al., 2019; Detert & Schmidt, 2021; Eißenberger et al., 2020), and since this strain allows a high standard of laboratory safety, we used it for the current study. In order to investigate the survival of C227/11Φcu in soil over time, we used a soil microenvironment model containing 25 g soil as described previously (Detert & Schmidt, 2021). The survival analysis was performed in autoclaved AL and non‐autoclaved AL soil samples. Autoclaved soil samples were inoculated with 10^9^ cfu/g soil, used for RNA isolation and to determine the viable counts during soil persistence. To obtain a sufficient amount of RNA, autoclaved AL was inoculated with a higher level of C227/11Φcu. Non‐autoclaved AL soil samples were only used for the survival analysis and were inoculated with 10^8^ cfu/g soil as described previously (Detert & Schmidt, 2021). The microenvironments were incubated at 4°C for 12 weeks and the viable counts (cfu/g soil) were analysed on selective TBX agar immediately after inoculation, and at 1, 4 and 12 weeks post inoculation. The viable counts of strain C227/11Φcu in autoclaved and non‐autoclaved soil microenvironments at 4°C are shown in Figure 1.

Incubation of *E. coli* O104:H4 C227/11Φcu in non‐autoclaved AL soil at 4°C resulted in a decrease of approximately 1.3 log cfu/g soil within 12 weeks. In comparison, only a slight decrease of approximately 0.1 log cfu/g soil was found for C227/11Φcu incubated in autoclaved AL samples at 4°C. These results let us suggest that the survival of *E. coli* O104:H4 C227/11Φcu was facilitated in autoclaved AL samples.

### Analysis of differentially expressed genes and gene sets

To further analyse the response and adaption of *E. coli* O104:H4 strain C227/11Φcu to components of the soil microenvironment, we performed differential transcriptomic analysis. For this purpose, the soil microenvironments consisting of autoclaved AL were additionally used for the isolation of RNA, immediately after inoculation at time point 0 (weeks), and after 1 and 4 weeks of incubation at 4°C. By performing gene expression analysis, we wanted to investigate genetic factors that were differentially regulated under the experimental conditions chosen. We were looking especially for regulated genes that are essential for adaption, survival and persistence to the soil environment, in a time‐dependent manner. Transcription patterns of the samples were analysed at time points 1 week and 4 weeks, and compared with those of time point 0. Gene expression analysis revealed that 1509 genes were significantly differentially expressed after persisting in soil for 1 week (Table S2). In more detail, 756 genes were downregulated and 753 genes were upregulated. After 4 weeks, 1507 genes were significantly differentially regulated (Table S3). Of these, 740 were downregulated and 767 upregulated.

**FIGURE 2:**
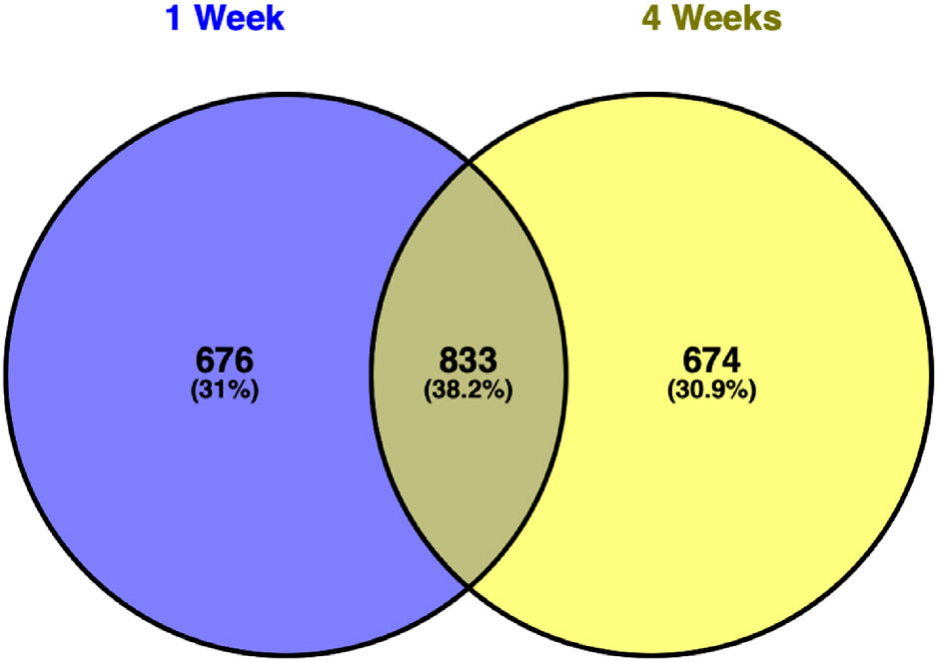
Venn diagram of the number of differentially expressed genes of C227/11Φcu in AL at 4°C after 1 and 4 weeks.

To visualize similarities and differences in the transcriptional pattern of strain C227/11Φcu in AL after 1 and 4 weeks, the number of similar and exclusive genes are illustrated in a Venn diagram in Figure 2. The Venn diagram in Figure 2 demonstrates that 833 genes were differentially expressed at both time points while 676 genes and 674 genes were exclusively differentially expressed after 1 and 4 weeks, respectively. We were especially interested in how the transcription pattern changed after 4 weeks to investigate adaption mechanisms for a long‐term survival in soil. After 4 weeks, we found that of the 674 exclusive genes, 330 were downregulated and 344 were upregulated (Table S6).

To get more insight into the regulated genes, we further analysed the most strongly regulated genes after 1 and 4 weeks in autoclaved AL (see Table 3). The *nar* operon (*narH*, *narI*, *narW*, *narZ*) was clearly upregulated at both time points with log_2_FC values between 4.64 and 7.53. Moreover, genes *narW and narZ* belong to the most strongly expressed genes after 1 and 4 weeks (Tables S1 and S2). In addition, genes encoding for different transporter and permeases (*actP*, *dppF*, *gabP*, *mglC*, *yjfF*), for L‐arabinose degradation (*araD*) and for D‐ribose phosphorylation (*rbsD*) were also found to be overexpressed at both time points. In contrast, 8 genes were only upregulated after 1 or 4 weeks. After 1 week, genes for 4‐aminobutyrate (GABA) uptake (*gabP*) and degradation (*gabT*) for succinate production were strongly upregulated with log_2_FC of 7.39 and 6.83, respectively. In addition, *hyi*, *gcI* and *glxR* which participate in the degradation of glycolate to generate pyruvate were exclusively upregulated after 1 week of incubation. Upregulation of zinc resistance sensor/chaperone ZraP was only found after 4 weeks. In addition, different DUF domain containing proteins mainly with unknown function were uniquely upregulated at this time point.

**TABLE 3:** Most significant upregulated genes of *Escherichia coli* O104:H4 C227/11Φcu in autoclaved AL at 4°C.

Gene name	Description	Log_2_FC^a^	
		1 week	4 weeks
AAF13_RS15145	YbgS‐like family protein	**n.d.e**	**5.16**
AAF13_RS18930	DUF2502 domain‐containing protein	**n.d.e**	**5.36**
AAF13_RS19105	DUF1328 domain‐containing protein	2.20	5.39
AAF13_RS19690	N‐acetylmannosamine‐6‐phosphate 2‐epimerase	3.03	5.32
AAF13_RS31850	DUF1471 domain‐containing protein	**n.d.e**	**6.20**
*actP*	Cation/acetate symporter ActP	6.73	2.17
*araD*	L‐ribulose‐5‐phosphate 4‐epimerase	8.45	2.78
*dppF*	Dipeptide ABC transporter ATP binding subunit DppF	7.12	2.03
*gabP*	GABA permease	7.39	1.87
*gabT*	4‐Aminobutyrate‐2‐oxoglutarate transaminase	**6.83**	**n.d.e**
*gcI*	Glyoxylate carboligase	**7.50**	**n.d.e.**
*glxR*	2‐Hydroxy‐3‐oxopropionate reductase	**7.78**	**n.d.e.**
*hyi*	Hydroxypyruvate isomerase	**7.63**	**n.d.e.**
*mglC*	Galactose/methyl galactoside ABC transporter permease MglC	6.63	2.24
*narH*	Nitrate reductase subunit beta	6.90	5.48
*narI*	Respiratory nitrate reductase subunit gamma	6.40	4.64
*narW*	Nitrate reductase molybdenum cofactor assembly chaperone	7.53	5.98
*narZ*	Nitrate reductase Z subunit alpha	6.23	5.27
*rbsB*	Ribose ABC transporter substrate‐binding protein RbsB	4.78	4.87
*rbsD*	D‐ribose pyranase	4.08	5.07
*rplS*	50S ribosomal protein L19	2.88	5.18
*yjfF*	Sugar ABC transporter permease YjfF	7.06	4.55
*zraP*	Zinc resistance sensor/chaperone ZraP	**n.d.e.**	**5.77**

*Note*: The 10 most highly upregulated genes in week 1 or week 4 are highlighted in grey. By this, differences and similarities between the two time points are demonstrated. Log_2_FC in bold are only upregulated in week 1 but not in week 4 or vice versa.

^a^ n.d.e., not differentially expressed.

**FIGURE 3:**
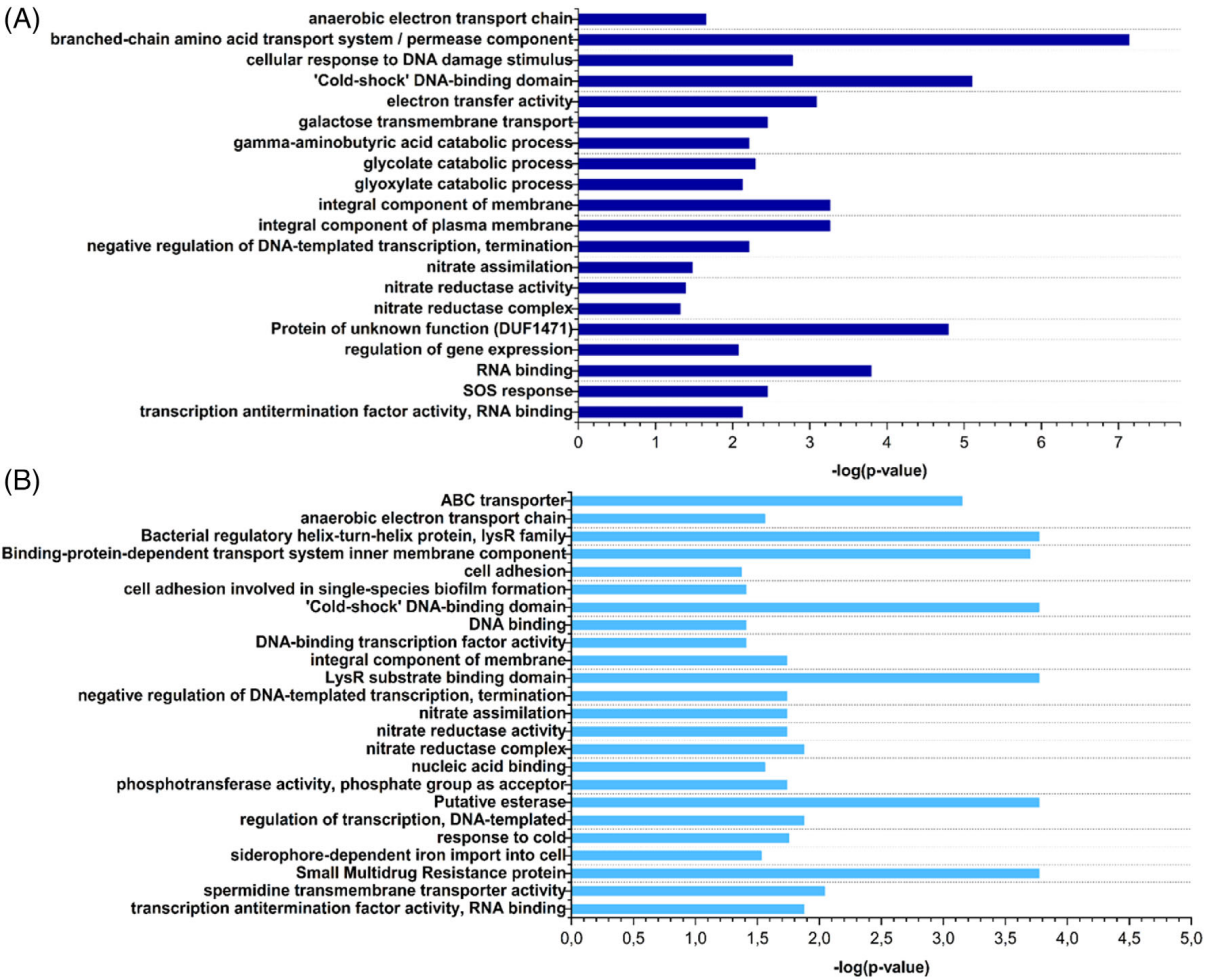
Gene set enrichment analysis of transcriptomic analysis of C227/11Φcu in AL soil at 4°C after (A) 1 week and (B) 4 weeks.

To further describe the transcriptional changes at both time points, gene set enrichment analysis was performed. By this method, significantly enriched or depleted classes of genes or proteins are identified to better understand the adaption mechanisms as well as biological processes required for soil survival. The results of the analysis are shown in Figure 3. The enrichment analysis of C227/11Φcu in AL at 4°C showed that the bacterial cells expressed significantly more genes for e.g. amino acid transport systems (ABC transporter), galactose transmembrane transport, glycolate and glyoxylate catabolic processes, nitrate reduction and SOS response, than the control sample at time point 0. Here, differences between 1 and 4 weeks are further demonstrated. After 4 weeks, genes for resistance proteins, RNA and DNA binding, cell adhesion and adhesion involved in biofilm formation were enriched. These enriched groups of up‐ or downregulated genes are investigated in more detail in the following sections.

### Regulation of stress response genes

For survival in the soil environment, *E. coli* must react to a variety of external stresses which include temperature changes, osmotic stress, oxidative stress, desiccation, and nutrient starvation (Battesti et al., 2011; Coldewey et al., 2007; Hryckowian & Welch, 2013; Stasic et al., 2012). The differential expression analysis revealed that C227/11Φcu increased the expression of different stress related genes. Table 4 shows some selected stress response genes which were significantly expressed after 1 and 4 weeks in autoclaved AL soil at 4°C.

**TABLE 4:** Stress associated genes of *Escherichia coli* O104:H4 C227/11Φcu expressed during persistence in autoclaved AL at 4°C after 1 and 4 weeks.

Stress response		Log_2_FC^a^	
Genes	Description	1 week	4 weeks
*bhsA*	Multiple stress resistance protein BhsA	−7.59	n.d.e.
*bsmA*	Biofilm peroxide resistance protein BsmA	2.96	3.01
*cspD*	Cold shock‐like protein CspD; DNA replication inhibitor	4.10	1.89
*pspA*	Phage shock protein PspA	−1.58	3.89
*pspB*	Envelope stress response membrane protein PspB	n.d.e.	3.94
*pspC*	Envelope stress response membrane protein PspC	n.d.e.	4.65
*pspD*	Phage shock protein PspD	n.d.e.	4.53
*pspF*	Phage shock protein operon transcriptional activator	−2.13	3.94
*pspG*	Envelope stress response protein PspG	n.d.e.	4.05
*rpoH*	RNA polymerase sigma factor RpoH	−4.61	n.d.e.
*rpoS*	RNA polymerase sigma factor RpoS	2.42	3.23
*uspA*	Universal stress protein UspA	1.36	2.34
*uspD*	Universal stress protein UspD	1.62	2.11

*Note*: Green, downregulated genes; blue, upregulated genes.

^a^ n.d.e., not differentially expressed.

The expression of the above‐mentioned genes, except of *rpoH* and *bhsA*, was highly upregulated and usually, the log_2_FC values increased with the persistence time in soil. The RNA polymerase sigma factor RpoH and the multiple stress resistance protein BhsA were downregulated after 1 week and no significant differential expression was found after 4 weeks. Nevertheless, the inoculation into the soil environment resulted in an increased expression of various stress factors. The *rpoS* gene is induced by different environmental stresses such as nutrient starvation and its expression was increased 5.4‐fold after 1 week (log_2_FC of 2.42) and 9.4‐fold after 4 weeks (log_2_FC of 3.23). In addition, we could assign many differentially expressed genes to the RpoS‐regulon (Dong & Schellhorn, 2009) (Tables S4 and S5). A further stress related gene, which was differentially expressed in C227/11Φcu during persistence in autoclaved AL was *cspD*. CspD is a toxin that is known to act as DNA replication inhibitor and that influences the persister cell formation. The expression is induced at stationary phase and upon glucose starvation. Nearly all of the genes were differentially expressed and showed the same trend after 1 and 4 weeks except of *pspB*, *pspC*, *pspD* and *pspF*. Differential expression analysis showed that the gene for the phage shock protein operon transcriptional activator (*pspF*) as well as genes that encode for two envelope stress response membrane proteins (*pspB* and *pspC*), were only upregulated after 4 weeks. The gene *pspD* encoding for a phage shock protein, was downregulated after 1 week and highly upregulated after 4 weeks in the soil environment. Further stress related genes that were exclusively expressed after 4 weeks were genes correlated with biofilm formation such as *bssR*. Further genes that correlate with envelope stress were *ldtA*, *ldtB*, *ldtD* and *ldtE*. These were upregulated exclusively after 4 weeks (Table S6). These genes encode L, D‐transpeptidases which contribute to the cell stability. In addition, we found that genes for biofilm formation were differentially expressed, such as the biofilm regulator gene *bssR*, which was 6.51‐fold overexpressed after 4 weeks (Tables S3 and S6).

### Metabolic processes

The exposure of *E. coli* O104:H4 str. C227/11Φcu to autoclaved AL resulted obviously in an intense adaption to the soil environment. Changes in the transcriptional profile were also found for different metabolic processes. Thereby, the increased expression of genes linked to the consumption of alternative C‐ and N‐sources was significant (Tables S2 and S3). We found an enrichment of genes for ABC transporters (e.g. amino acid uptake) and genes correlated with glycolate and glyoxylate catabolic processes as well as with nitrate reduction. In addition, many genes linked to the β‐oxidation of fatty acids, uptake and metabolism of polyamines, and metabolism of amino acids such as L‐serine, were significantly differentially expressed.

#### Uptake, biosynthesis and degradation of amino acids

The enrichment analysis revealed that genes for amino acid transport systems were highly upregulated. Table 5 summarizes the upregulated genes correlated with amino acid transport from the environment into the bacterial cell. The genes for amino acid uptake were predominantly upregulated after 1 week. Only two genes (*metQ* and *yecC*) were found to be significantly upregulated also after 4 weeks. All other genes were not differentially expressed at the second time point. We found the same trend when looking at genes for amino acid biosynthesis. The results are summarized in Table S7. To correlate the uptake and synthesis of amino acids, we determined the concentration of essential amino acids in alluvial loam and autoclaved alluvial loam (Core Facility, University of Hohenheim). The results are shown in Table 6.

**TABLE 5:** Differentially expressed genes for amino acid transport of *E. coli* O104:H4 C227/11Φcu during persistence in autoclaved AL at 4°C after 1 and 4 weeks.

Amino acid transport		Log_2_FC^a^	
Genes	Description	1 week	4 weeks
*glnP*	Glutamine ABC transporter permease GlnP	2.23	n.d.e.
*hisP*	Histidine ABC transporter ATP‐binding protein HisP	1.84	n.d.e.
*metQ*	Methionine ABC transporter substrate‐binding lipoprotein MetQ	1.78	1.20
*yecC*	L‐cystine ABC transporter ATP‐binding protein YecC	2.61	2.40
*argT*	Lysine/arginine/ornithine ABC transporter substrate‐binding protein ArgT	2.88	n.d.e.
*artJ*	Arginine ABC transporter substrate‐binding protein	2.86	n.d.e.
*artM*	Arginine ABC transporter permease ArtM	2.55	n.d.e.
*artP*	Arginine ABC transporter ATP‐binding protein ArtP	2.09	n.d.e.
*artQ*	Arginine ABC transporter permease ArtQ	3.34	n.d.e.
*cycA*	D‐serine/D‐alanine/glycine transporter	1.53	n.d.e.
*sstT*	Serine/threonine transporter SstT	2.07	n.d.e.

*Note*: Green, downregulated genes; blue, upregulated genes.

^a^ n.d.e., not differentially expressed.

**TABLE 6:** Amino acid analysis of alluvial loam.

Amino acid	Concentration (mg/g AL)
Alanine	0.2
Arginine	0.1
Aspartic acid	0.6
Cysteine	0.1
Glutamic acid	0.5
Glycine	0.4
Histidine	0.1
Isoleucine	0.1
Leucine	0.2
Lysine	0.2
Methionine	0.04
Phenylalanine	0.2
Proline	0.2
Serine	0.2
Threonine	0.2
Tryptophan	<0.1
Tyrosine	0.04
Valine	0.2

Except of tryptophan, methionine and tyrosine, all other amino acids are present in concentrations >0.1 mg/g. Thus, the genes for uptake of tryptophan, methionine and tyrosine are not upregulated compared to the other amino acids (Table 5). Consequently, the biosynthesis of tryptophan was highly upregulated after 1 weeks in autoclaved AL (Table S7). The analysis further showed that the degradation of amino acids was highly upregulated after 1 week of incubation. In addition, the transcriptomic analysis revealed that amino acids were used by *E. coli* O104:H4 str. C227/11Φcu as alternative C‐ and N‐source (Table 7).

**TABLE 7:** Differential expressed genes involved in amino acid degradation for generation of carbon or nitrogen sources.

Amino acid degradation			Log_2_FC^a^	
Genes	Gene name	Description	1 week	4 weeks
*tnaA*	Tryptophanase	*L‐tryptophan → ammonium + indole + pyruvate*	3.00	n.d.e.
*aspA*	Aspartate ammonia‐lyase	*L‐aspartate → fumarate + ammonium*	2.63	1.55
*ldcC*	Lysine decarboxylase	*L‐lysine + H* ^*+*^ *→ CO*_*2*_ *+ cadaverine*	1.50	2.72
*speA*	Biosynthetic arginine decarboxylase	*L‐arginine → putrescine*	1.74	1.62
*sdaA*	L‐serine ammonia‐lyase	*L‐serine → pyruvate + ammonium*	n.d.e.	1.63
*sdaB*	L‐serine ammonia‐lyase II		2.01	n.d.e.

*Note*: Green, downregulated genes; blue, upregulated genes.

^a^ n.d.e., not differentially expressed.

**FIGURE 4:**
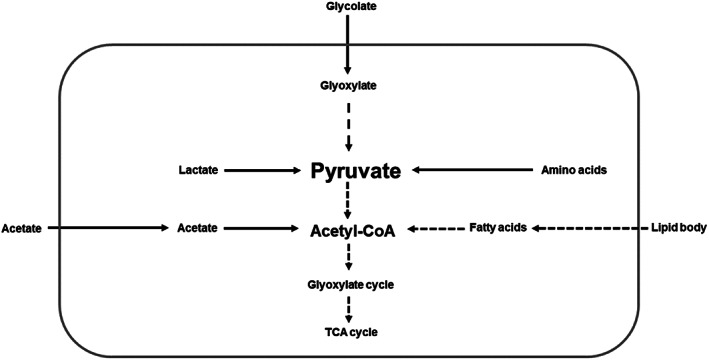
Upregulated genes for pyruvate and acetyl‐CoA generation from different carbon sources present in autoclaved AL.

We further asked which metabolic processes are required for the long‐term survival of *E. coli* O104:H4 str. C227/11Φcu in agricultural soil. The gene set enrichment analysis revealed that glycolate and glyoxylate catabolic processes are important as well as nitrate reduction (Figure 3). Highly upregulated pathways for pyruvate and Acetyl‐CoA generation are depicted in Figure 4.

With the expression analysis, we found an upregulation of genes linked to the consumption of acetate, lactate, glycolate, fatty acids and amino acids as carbon source for energy generation. The degradation of amino acids is shown in Table 7 while the genes with corresponding log_2_FC that are correlated to the other pathways are given in Table S8.

**FIGURE 5:**
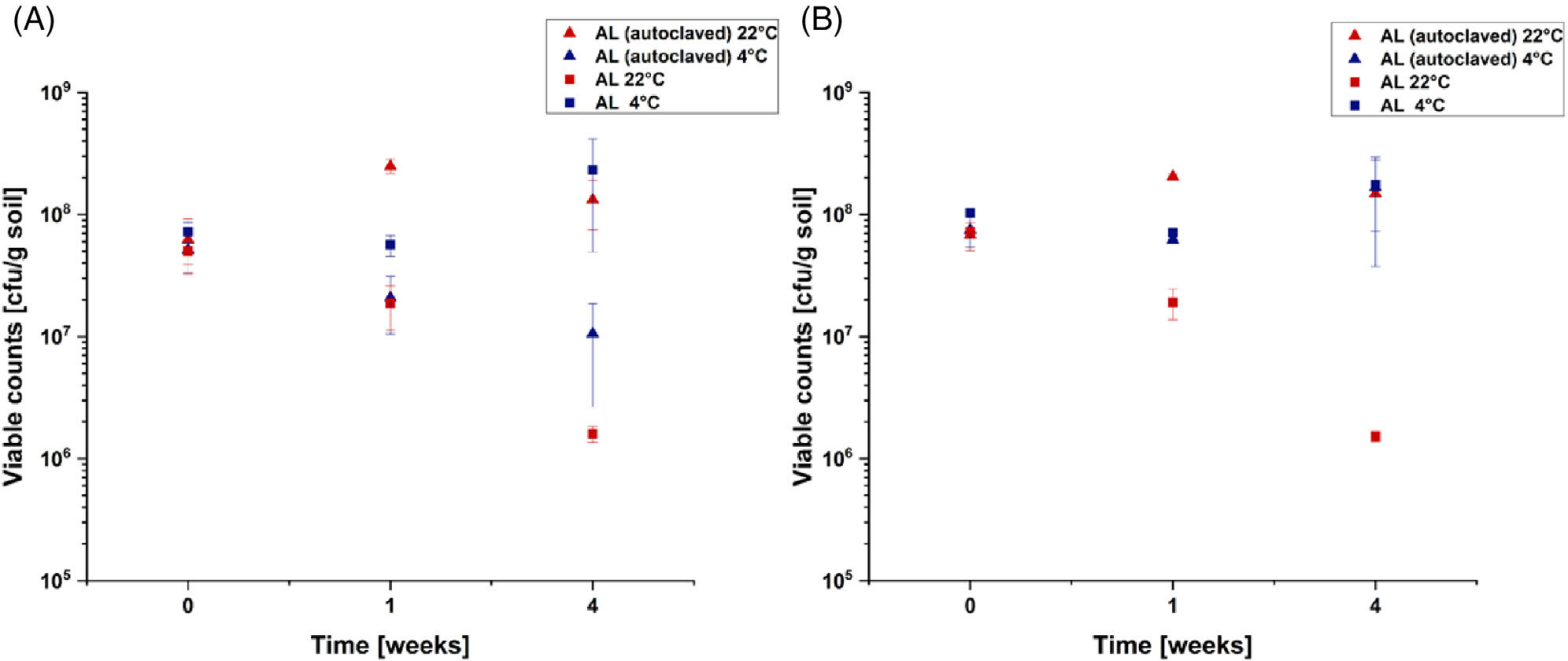
Survival of (A) *Escherichia coli* O104:H4 strain C227/11Φcu and (B) its isogenic *acs* deletion mutant in soil microenvironments consisting of 25 g autoclaved AL and non‐autoclaved AL soil at 4°C and 22°C (as indicated). The soil was inoculated with 10^8^ (non‐autoclaved) cfu/g soil and incubated for 4 weeks. The experiment was performed in two biological duplicates. Error bars show the standard deviation of a biological replicate.

In our data, we found that genes for the uptake of acetate (*actP*) and for the conversion of acetate to acetyl‐CoA (*acs* and *ackA*) are highly upregulated after 1 and 4 weeks (Table S8). Based on that we hypothesized that acetate is an important carbon source in AL and that the persistence is reduced by deletion of *acs*. For this purpose, we prepared an isogenic deletion mutant of *E. coli* O104:H4 str. C227/11Φcu. The successful construction was verified by PCR (Figure S1) and the isogenic deletion strain was used to analyse the persistence in the soil microenvironments compared to the wild type strain. Soil samples of AL and autoclaved AL were inoculated with 10^8^ cfu/g of *E. coli* O104:H4 str. C227/11Φcu or its corresponding *acs* deletion mutant. The samples were either incubated at 22°C or 4°C and the cfu number was determined at the day of inoculation and 1 and 4 weeks post inoculation. The results of all four temperature/soil combinations are demonstrated in Figure 5.

Here, we did not find any differences in soil survival between C227/11Φcu (Figure 5A) and the *acs* deletion mutant (Figure 5B). If detected, the reduction in viable counts was identical for all tested conditions. During the 4 weeks of incubation, reduction was only found for non‐autoclaved AL at 22°C. Since we did not find any difference between wild type and mutant strain, we concluded that the deletion did not affect the soil persistence of C227/11Φcu.

In addition to acetate conversion, the overexpression of genes linked to the glyoxylate cycle, to glycolate degradation and to the β‐oxidation of fatty acids are very remarkable at both time points (Table S8). Moreover, lactate degradation seems to be an important C‐source after 1 week of incubation. After 4 weeks, the genes for lactate uptake and conversion to pyruvate were not differentially expressed. Consequently, different genes for further carbon sources were upregulated exclusively after 4 weeks. Here, genes for L‐ascorbate degradation such as *ulaA* (log_2_FC = 3.19) and *ulaG* (log_2_FC = 3.22) were upregulated. In addition, *xylB*, a xylulokinase, which participates in the degradation of D‐xylulose was upregulated (log_2_FC = 1.80) after 4 weeks (Table S3). The comparison of the two time points highlighted that genes for amino acid uptake (Table 5) and degradation (Table 6) as well as the use of lactate or glycolate as C‐source were mostly not differentially expressed after 4 weeks. At this time point, we found that the upregulation of genes correlated with chemotaxis and motility. These genes are shown in Table S9.

**FIGURE 6:**
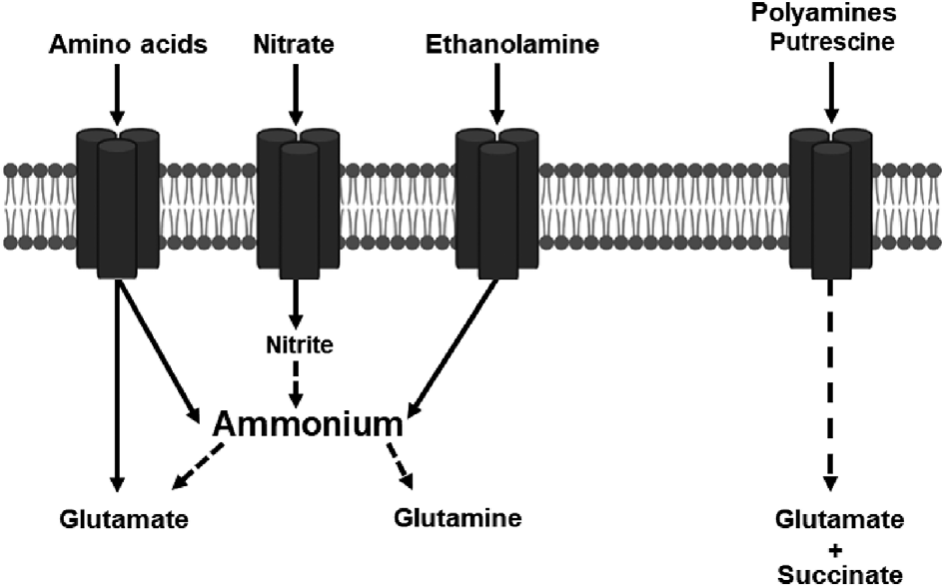
Schematically illustrated pathways of nitrogen sources that were found to be upregulated in *Escherichia coli* C227/11Φcu. Genes for generation of ammonium or glutamate from amino acids, nitrate, ethanolamine and polyamine were upregulated.

Besides carbon, nitrogen is a key nutrient which is required for bacterial growth. It is an essential component for, amino acids, coenzymes, nucleic acids and polyamines. Genes correlating with the generation of nitrogen are shown in Table S10 and are schematically demonstrated in Figure 6.

The genes of the *nar* operon were highly upregulated at both time points with log_2_FC up to 7.53 (Table S10). By this, nitrate is reduced to nitrite which can be further converted to ammonium (Figure 6). Further nitrogen sources are amino acids (see Table 7), ethanolamine and polyamines as illustrated in Figure 6. We also found an increased expression of genes (*eutA*, *eutB*, *eutC*, *eutK*, *eutL*) for conversion of ethanolamine to ammonium and acetaldehyde (Table S10). Transport systems for the uptake of polyamines such as putrescine or spermidine were highly upregulated as well as genes for degradation of putrescine (*puuE*). Putrescine is firstly converted to 4‐aminobutyrate (GABA) which can be further metabolized to glutamate and succinate. In addition, we found a remarkable upregulation of the GABA permease (*gabP*) especially after 1 week with a log_2_FC value of 7.39 (Table 3).

## DISCUSSION

Since we could show in a recent study that *E. coli* O104:H4 strain C227/11Φcu was able to survive for up to 12 weeks in soil microenvironments (Detert & Schmidt, 2021), we consequently were interested in the intrinsic genetic factors facilitating survival. Differential genomic analysis which differentiated between the gene expression state at the time point of inoculation and time points 1 and 4 weeks after inoculation showed significant differences. Although alluvial loam is a rich agricultural soil, it can be considered as a poor substrate when compared to those present in the human or animal gut. In a soil environment, *E. coli* needs to adapt to a low nutrient availability. We used autoclaved alluvial loam which is largely reduced in soil microbial community. Thus, we must consider that the differences in gene expression pattern might differ to natural conditions. In this study, we focused on changes in stress response and metabolic activity which enable the survival of this enteric pathogen in sublethal environments.

By differential expression analysis, we found the upregulation of different stress related genes. In a previous study, we showed that the deletion of *rpoS* resulted in a significant reduction of survival ability of C227/11Φcu in agricultural soil samples (Detert & Schmidt, 2021). In this study, we found the upregulation of *rpoS* after 1 and 4 weeks in AL by 5.4 and 9.4‐fold, respectively. The sigma factor RpoS is a conserved stress regulator that regulates around 10% of the genes of the *E. coli* genome, which include approximately 500 genes (Weber et al., 2005). The differential expression of RpoS‐dependent genes was investigated in the present study. Dong and Schellhorn (2009) showed that the deletion of *rpoS* had a pronounced effect on genomic expression of *E. coli* O157:H7 strain EDL933 in the stationary growth phase. The authors found that more than 1000 genes were differentially expressed in the stationary phase as response to *rpoS* deletion and summarized the top 100 most RpoS‐dependent genes. Of these 100 described genes, we found 50 genes, which were differentially expressed after 1 week and 41 differentially expressed genes after 4 weeks (Tables S4 and S5). RpoS is the master regulator of the general stress response in *E. coli* and essential for survival under a variety of stress conditions (Hengge, 2009). The importance of a functional RpoS for long‐term soil survival of *E. coli* has also been shown by other authors (Somorin et al., 2016, 2017; Somorin & O'Byrne, 2017). The authors demonstrated that the *rpoS* gene is highly conserved in long‐term soil persistent *E. coli* strains and that RpoS is essential for protecting the bacteria against unfavourable conditions such as low pH or reduced moisture which are present in soils. For survival in the soil environment, *E. coli* must react to much more external stresses which include temperature changes, osmotic stress, oxidative stress, desiccation, and nutrient starvation (Battesti et al., 2011; Coldewey et al., 2007; Hryckowian & Welch, 2013; Stasic et al., 2012). A further stress related gene, which was differentially expressed in C227/11Φcu during persistence in autoclaved AL was *cspD*. CspD is a toxin that is known to act as DNA replication inhibitor and that influences the persister cell formation. The expression is induced at stationary phase and upon glucose starvation (Yamanaka et al., 2001). Overall, the introduction into the soil environment resulted in an increased expression of various stress related factors (Table 4). This was also shown in a study of Brennan et al. (2013). The authors compared the protein expression of an environmental *E. coli* isolate with the laboratory *E. coli* strain K12 at 15°C. The environmental isolate showed a nutritional flexibility and expression of proteins correlated with cold adaption while *E. coli* K12 was found to express higher levels of stress‐related proteins (Brennan et al., 2013). In general, the upregulation of different stress response mechanisms allow *E. coli* strains to adapt to unfavourable conditions and to nutrient starvation. The survival ability in AL was analysed in previous studies (Detert & Schmidt, 2021; Eißenberger et al., 2020). In the current study, we again analysed the survival of C227/11Φcu in AL and compared it to the survival in autoclaved AL samples. A study of Baker et al. (2021) already demonstrated that the survival of *E. coli* O157:H7 was enhanced in heat treated soil samples. The authors correlated this with a reduction in the antagonistic soil microbiota. Similar observations were found in the current study. The incubation of C227/11Φcu in AL at 4°C for 12 weeks indicated only slight differences of 1 log units between autoclaved and non‐autoclaved samples (Figure 1). The incubation of C227/11Φcu in autoclaved AL soil at 22°C clearly indicated the enhanced survival compared to untreated soil samples (Figure 3). Here, a decrease in bacterial cfu counts was observed in AL at 22°C within 4 weeks, while no reduction was observed in autoclaved samples. We even saw an increase in viable counts within the first week of incubation. These finding let us hypothesize that C227/11Φcu can utilize the nutrient sources present in AL. Of course, carbohydrates vary in presence and concentration, but soil is usually known to show glucose‐limited conditions (Kalbitz et al., 2000; NandaKafle et al., 2018). Thus, we looked for changes in the metabolic processes during soil persistence and found differential expression of genes correlated with different uptake mechanisms as well as with the consumption of alternative C‐ and N‐sources.

The enrichment analysis revealed that genes for amino acid transport systems as well as for biosynthesis were highly upregulated after 1 week. The analysis for amino acid concentrations in autoclaved AL correlated amino acid transport genes with those of biosynthesis. Genes for uptake of amino acids such as tryptophan that are present in concentrations <0.1 mg/g were not differentially expressed. Consequently, the biosynthesis of tryptophan was highly upregulated. A recent study showed that genes for tryptophan biosynthesis were also upregulated in *Salmonella enterica* during incubation in a soil environment (Schierstaedt et al., 2020). Hamilton et al. (2009) correlated tryptophan metabolism with biofilm formation in *Salmonella*. Similar results were found for *E. coli* where the tryptophanase (*tna*) operon is associated with biofilm formation (Di Martino et al., 2002; Di Martino et al., 2003). Further transcriptomic studies demonstrated that biofilm growth of *E. coli* leads to the upregulation of genes required for amino acid metabolism as well as for stress response and anaerobic respiration (Ren et al., 2004; Schembri et al., 2003). A study of Tao et al. (1999) showed that the upregulation of genes for amino acid biosynthesis are induced during growth in low nutrient environments. The transcriptomic analysis of *S*. *enterica* in soil revealed the enrichment of genes correlated with amino acid biosynthesis (Schierstaedt et al., 2020). We also found the upregulation of many amino acid biosynthesis genes. By this, *E. coli* C227/11Φcu might adapt to unfavourable conditions of alluvial loam. The transcriptomic analysis further highlighted that amino acids were metabolized by *E. coli* O104:H4 str. C227/11Φcu and used as alternative N‐source (Table 7). Genes for deamination of amino acids were upregulated. By this, ammonium is generated that is further assimilated to glutamate. Glutamate is the major intracellular nitrogen donor and precursor for amino acids or polyamines (Helling, 1998). Nitrogen is a key nutrient which is required for the bacterial growth and represents an essential component for example, amino acids, coenzymes, nucleic acids and polyamines. Further available nitrogen sources in AL seem to be nitrate, ethanolamine and polyamines. Genes of the *nar* operon were highly upregulated after 1 and 4 weeks indicating that nitrate represents an important source for nitrogen. Nar only plays a role when nitrate is present in the environment. By this, expression of genes for nitrate reduction is activated (Matsuoka & Shimizu, 2011). Nitrate occurs naturally in soil and is introduced, for example, via fertilizer application or decomposing of plant residues on the field. *E. coli* can reduce nitrate to nitrite which is further converted to ammonium.

One further adaption to the soil environment is the use of alternative N‐sources such as polyamines or ethanolamines. Metabolism of bacterial polyamines is on the one hand correlated with low nitrogen availability and on the other hand important for the survival under high concentrations of these compounds. In *E. coli*, polyamines can be either taken up from the environment or synthesized from the amino acids methionine, ornithine, lysine, and arginine (Gevrekci, 2017; Miller‐Fleming et al., 2015). The concentration of putrescine in soil was found to be between 0.28 and 0.56 nmol/g (Young & Chen, 1997). In the transcriptomic data, we found the upregulation of genes for putrescine uptake and biosynthesis. The changes in metabolism were investigated in previous studies and correlated with anaerobically growth conditions. The study of Chattopadhyay et al. (2009) demonstrated that polyamines are required for the growth under anaerobic conditions. Moreover, polyamines are known to be involved in nucleic acid and protein biosynthesis and structure (Tabor & Tabor, 1985). Furthermore, polyamines play a role in biofilm formation and response to oxidative stress (Nesse et al., 2015; Wortham et al., 2007).

Nitrogen content as well as the availability of carbon sources are critical factors that affects the persistence of *E. coli* under unfavourable conditions present in the soil environment. Genes for acetate uptake as well as genes involved in glyoxylate cycle were enriched after 1 week of incubation in AL. The glyoxylate cycle is a modified version of the TCA cycle and only induced during growth with simple carbon compounds such as acetate. The cycle is repressed, if glucose or more complex C‐sources such as cellulose are available. *E. coli* K12 which was grown in minimal glucose medium expressed more genes correlated with D‐lactate utilization, acetate formation and acetate utilization. Once the available glucose was consumed, the cells switched to metabolism of acetate. By this, genes for TCA cycle and the glyoxylate bypass were upregulated (Tao et al., 1999). Schierstaedt et al. (2020) also found the upregulation of genes involved in the glyoxylate cycle when *S. enterica* was exposed to soil environments. By the metabolism of acetate and other C‐sources such as lactate, fatty acids or glycolate, adaption and persistence of C227/11Φcu in the soil microenvironments was possible. Although our study has a descriptive nature, the data showed very clearly that C227/11Φcu responds to the soil environment by changing its transcriptional profile regarding for example stress response and basic metabolic processes.

## CONCLUSION

The results of this study have shown that pathogenic *E. coli* strain C227/11Φcu is able to adapt to the environment of a typical agricultural soil at low temperature and can manage low nutrient availability under high stress conditions. Although intensive growth is not expected at this temperature, the study shows a huge impact on global gene transcription. Uptake of amino acids and metabolism of plant‐derived sugars such as ribose seems to be common. We did not observe the upregulation of strain specific virulence factors. Moreover, general metabolic pathways that are present in most if not all *E. coli* play a role for survival and persistence. To investigate this hypothesis, transcriptome analysis of different serotypes of pathogenic *E. coli* should be analysed in future studies.

## AUTHOR CONTRIBUTIONS

**Katharina Detert:** Conceptualization (lead); formal analysis (lead); investigation (lead); methodology (lead); validation (lead); visualization (equal); writing – original draft (lead); writing – review and editing (equal). **Jonathan Währer:** Data curation (lead); formal analysis (equal); methodology (equal); software (lead); writing – review and editing (supporting). **Kay Nieselt:** Conceptualization (equal); data curation (lead); formal analysis (equal); methodology (lead); software (equal); supervision (equal); writing – review and editing (equal). **Herbert Schmidt:** Conceptualization (equal); funding acquisition (lead); project administration (equal); resources (lead); supervision (lead); writing – review and editing (equal).

## CONFLICT OF INTEREST STATEMENT

None of the authors declare any conflicts.

## Supporting information


**Table S7.** Differentially expressed genes of C227/11Φcu incubated in AL at 4°C that are associated with amino acid biosynthesis.
**Table S8.** Differentially expressed genes of C227/11Φcu incubated in AL for up to 4 weeks at 4°C that are associated with the metabolism of alternative C‐sources.
**Table S9.** Differentially expressed genes of C227/11Φcu incubated in AL for up to 4 weeks at 4°C that are associated with the chemotaxis and motility.
**Table S10.** Differentially expressed genes of C227/11Φcu incubated in AL for up to 4 weeks at 4°C that are associated with the metabolism of alternative N‐sources.
**Figure S1.** Agarose gel electrophoresis to confirm the incorporation and subsequent deletion of kanamycin resistance cassette to construct isogenic *acs* deletion mutant of C227/11Φcu.

**Table S1.** Overview of total reads and reads mapped to *E. coli* genome of the analysed samples.

**Table S2.** Significantly expressed genes of *E. coli* O104:H4 C227/11Φcu in alluvial loam at 4°C after 1 week.

**Table S3.** Significantly expressed genes of *E. coli* O104:H4 C227/11Φcu in alluvial loam at 4°C after 4 weeks.

**Table S4.** RpoS‐dependent genes of *E. coli* O104:H4 C227/11Φcu differentially expressed in alluvial loam at 4°C after 1 week.

**Table S5.** RpoS‐dependent genes of *E. coli* O104:H4 C227/11Φcu differentially expressed in alluvial loam at 4°C after 4 weeks.

**Table S6.** Exclusive differentially expressed genes of *E. coli* O104:H4 C227/11Φcu in alluvial loam at 4°C after 4 weeks.

## Data Availability

The data that supports the findings of this study are available in the supplementary material of this article, and in NCBI's Gene Expression Omnibus (GEO) under the accession no. GSE222589. The bacterial strains are available from our laboratory upon request.
