# Supplementary material for: Broad time‐dependent transcriptional activity of metabolic genes of E. coli O104:H4 strain C227/11Φcu in a soil microenvironment at low temperature
Source: Environ Microbiol Rep. 2023 Aug 29;15(6):582–96. doi: 10.1111/1758-2229.13198 (PMC10667640; doi:10.1111/1758-2229.13198)
Supplement: Supplementary file 1 — Table S7. Differentially expressed genes of C227/11Φcu incubated in AL at 4°C that are associated with amino acid biosynthesis. Table S8. Differentially expressed genes of C227/11Φcu incubated in AL for up to 4 weeks at 4°C that are associated with the metabolism of alternative C‐sources. Table S9. Differentially expressed genes of C227/11Φcu incubated in AL for up to 4 weeks at 4°C that are associated with the chemotaxis and motility. Table S10. Differentially expressed genes of C227/11Φcu incubated in AL for up to 4 weeks at 4°C that are associated with the metabolism of alternative N‐sources. Figure S1. Agarose gel electrophoresis to confirm the incorporation and subsequent deletion of kanamycin resistance cassette to construct isogenic acs deletion mutant of C227/11Φcu. [file EMI4-15-582-s005.docx]

**Supplemental Material to Manuscript:

Survival of *E. coli* O104:H4 Strain C227/11Φcu in a Soil Microenvironment is Associated with a Broad Time-Dependent Τranscriptional Activity of Metabolic Genes**

**Katharina Detert, Jonathan Währer, Kay Nieselt and Herbert Schmidt**

**Table S7.** Differentially expressed genes of C227/11Φcu incubated in AL at 4 °C that are associated with amino acid biosynthesis.

| **Amino acid biosynthesis** | |  | ***Log_2_FC** | |
| --- | --- | --- | --- | --- |
| **Genes** | **Gene name** | **Description** | **1 week** | **4 weeks** |
| *aspA* | aspartate ammonia-lyase | *L-aspartate degradation to L-glutamine* | **2.63** | **1.55** |
|  |  |  |  |  |
| *gltB* | glutamate synthase large subunit | *L-glutamine degradation to L-glutamate* | **4.39** | **n.d.e.** |
| *gltD* | glutamate synthase subunit GltD |  | **5.77** | **1.68** |
|  |  |  |  |  |
| *argA* | amino-acid N-acetyltransferase | *L-glutamate degradation to L-ornithine and  L-arginine* | **6.09** | **3.42** |
| *argB* | acetylglutamate kinase |  | **2.57** | **n.d.e.** |
| *argD* | bifunctional acetylornithine/succinyldiaminopimelate transaminase |  | **4.35** | **2.02** |
| *argE* | acetylornithine deacetylase |  | **2.93** | **2.48** |
| *argF* | ornithine carbamoyltransferase |  | **3.86** | **n.d.e.** |
| *argG* | argininosuccinate synthase |  | **1.78** | **n.d.e.** |
| *argH* | argininosuccinate lyase |  | **3.37** | **n.d.e.** |
|  |  |  |  |  |
| *astA* | arginine N-succinyltransferase | *L-arginine degradation (AST pathway) to L-glutamate and succinate* | **1.93** | **1.97** |
| *astB* | N-succinylarginine dihydrolase |  | **1.70** | **1.81** |
| *astD* | succinylglutamate-semialdehyde dehydrogenase |  | **2.33** | **2.29** |
| *astE* | succinylglutamate desuccinylase |  | **2.60** | **1.99** |
|  |  |  |  |  |
| *lysC* | lysine-sensitive aspartokinase 3 | *L-aspartate degradation to L-threonine* | **2.80** | **n.d.e.** |
| *metL* | bifunctional aspartate kinase/homoserine dehydrogenase II |  | **1.47** | **n.d.e.** |
| *asd* | threonine synthase |  | **1.75** | **n.d.e.** |
|  |  |  |  |  |
| *lysC* | lysine-sensitive aspartokinase 3 | *L-lysine biosynthesis from L-aspartate* | **2.80** | **n.d.e.** |
| *asd* | aspartate-semialdehyde dehydrogenase |  | **1.75** | **n.d.e.** |
| *argD* | bifunctional acetylornithine/succinyldiaminopimelate transaminase |  | **4.35** | **2.02** |
| *thrB* | homoserine kinase | *L-threonine biosynthesis* | **1.78** | **n.d.e.** |
| *thrC* | aspartate-semialdehyde dehydrogenase |  | **2.84** | **n.d.e.** |
|  |  |  |  |  |
| *tdh* | threonine dehydrogenase | *L-threonine degradation for glycine synthesis* | **3.39** | **1.49** |
| *kbI* | glycine C-acetyltransferase |  | **2.14** | **n.d.e.** |
|  |  |  |  |  |
| *ilvA* | threonine ammonia-lyase, biosynthetic | *L-threonine degradation for L-isoleucine synthesis* | **5.09** | **2.07** |
| *ilvB* | acetolactate synthase large subunit |  | **2.14** | **n.d.e.** |
| *ilvD* | dihydroxy-acid dehydratase |  | **3.46** | **n.d.e.** |
| *ilvE* | branched-chain-amino-acid transaminase |  | **2.19** | **n.d.e.** |
| *ilvI* | acetolactate synthase 3 large subunit |  | **1.61** | **1.66** |
| *ilvM* | acetolactate synthase 2 small subunit |  | **3.00** | **n.d.e.** |
| *ilvN* | acetolactate synthase small subunit |  | **4.10** | **2.25** |
|  |  |  |  |  |
| *^1^alaC* | alanine transaminase | *Synthesis of L-alanine via pyruvate^1^ and  L-valine^2^* | **3.58** | **n.d.e.** |
| *^2^avtA* | valine--pyruvate transaminase |  | **1.89** | **n.d.e.** |
|  |  |  |  |  |
| *leuA* | 2-isopropylmalate synthase | *Synthesis of L-leucine* | **1.18** | **n.d.e.** |
| *leuB* | 3-isopropylmalate dehydrogenase |  | **2.47** | **n.d.e.** |
| *leuC* | 3-isopropylmalate dehydratase large subunit |  | **4.29** | **1.78** |
| *leuD* | 3-isopropylmalate dehydratase small subunit |  | **4.51** | **2.30** |
|  |  |  |  |  |
| *metH* | methionine synthase | *L-methionine biosynthesis* | **2.02** | **n.d.e.** |
|  |  |  |  |  |
| *trpA* | tryptophan synthase subunit alpha | *L-tryptophan biosynthesis* | **3.45** | **n.d.e.** |
| *trpB* | tryptophan synthase subunit beta |  | **2.92** | **n.d.e.** |

*n.d.e = not differentially expressed

**Table S8.** Differentially expressed genes of C227/11Φcu incubated in AL for up to 4 weeks at 4 °C that are associated with the metabolism of alternative C-sources.

|  | | ***Log_2_FC** | |
| --- | --- | --- | --- |
| **Genes** | **Description** | **1 week** | **4 weeks** |
| **Acetate conversion to acetyl-CoA** | |  |  |
| *actP* | acetate symporter ActP | **6.73** | **2.17** |
| *acs* | acetate--CoA ligase | **5.39** | **2.12** |
| *ackA* | acetate kinase | **3.25** | **2.96** |
| **Glyoxylate cycle** | |  |  |
| *aceA* | isocitrate lyase | **4.90** | **3.37** |
| *aceB* | malate synthase A | **3.24** | **2.06** |
| *aceK* | bifunctional isocitrate dehydrogenase kinase/phosphatase | **4.63** | **2.77** |
| **Glycolate and glyoxylate degradation** | |  |  |
| *glcA* | glycolate permease GlcA | **6.48** | **n.d.e.** |
| *glcB* | malate synthase G | **4.82** | **1.47** |
| *glcD* | glycolate oxidase subunit GlcD | **3.28** | **n.d.e.** |
| *glcE* | glycolate oxidase subunit GlcE | **3.69** | **n.d.e.** |
| *glcF* | glycolate oxidase subunit GlcF | **4.18** | **1.38** |
| *gcI* | glyoxylate carboligase | **7.50** | **n.d.e.** |
| *glxR* | 2-hydroxy-3-oxopropionate reductase | **7.78** | **n.d.e.** |
| *garK* | glycerate 2-kinase | **3.54** | **n.d.e.** |
| **β-oxidation of fatty acids** | |  |  |
| *fadA* | acetyl-CoA C-acyltransferase FadA | **5.51** | **2.25** |
| *fadB* | fatty acid oxidation complex subunit alpha FadB | **5.53** | **2.26** |
| *fadD* | long-chain-fatty-acid--CoA ligase FadD | **1.56** | **1.14** |
| *fadE* | acyl-CoA dehydrogenase FadE | **2.82** | **n.d.e.** |
| *fadH* | NADPH-dependent 2,4-dienoyl-CoA reductase | **3.91** | **2.12** |
| *fadI* | acetyl-CoA C-acyltransferase FadI | **1.28** | **n.d.e.** |
| *fadJ* | fatty acid oxidation complex subunit alpha FadJ | **3.27** | **2.15** |
| *fadL* | long-chain fatty acid transporter FadL | **5.27** | **1.79** |
| **Lactate degradation** | |  |  |
| *lldD* | quinone-dependent L-lactate dehydrogenase | **5.58** | **n.d.e.** |
| *lldP* | L-lactate permease | **4.82** | **n.d.e.** |

*n.d.e = not differentially expressed

**Table S9.** Differentially expressed genes of C227/11Φcu incubated in AL for up to 4 weeks at 4 °C that are associated with the chemotaxis and motility.

| **Genes** | **Description** | ***Log_2_FC  after 4 weeks** |
| --- | --- | --- |
| *motA* | flagellar motor stator protein MotA | **2.84** |
| *motB* | flagellar motor protein MotB | **3.65** |
| *cheA* | chemotaxis protein CheA | **3.36** |
| *cheW* | chemotaxis protein CheW | **2.15** |
| *fliA* | RNA polymerase sigma factor FliA | **2.69** |

*n.d.e = not differentially expressed

**Table S10.** Differentially expressed genes of C227/11Φcu incubated in AL for up to 4 weeks at 4 °C that are associated with the metabolism of alternative N-sources.

|  | | ***Log_2_FC** | |
| --- | --- | --- | --- |
| **Genes** | **Description** | **1 week** | **4 weeks** |
| **Nitrate reduction to nitrite** | |  |  |
| *narH* | nitrate reductase subunit beta | **6.90** | **5.49** |
| *narI* | respiratory nitrate reductase subunit gamma | **6.40** | **4.64** |
| *narL* | two-component system response regulator NarL | **1.50** | **2.34** |
| *narU* | nitrate/nitrite transporter NarU | **5.14** | **4.86** |
| *narW* | nitrate reductase molybdenum cofactor assembly chaperone | **7.53** | **5.98** |
| *narZ* | nitrate reductase Z subunit alpha | **6.23** | **5.27** |
| **Reduction of ethanolamine** | |  |  |
| *eutA* | ethanolamine ammonia-lyase reactivating factor EutA | **2.93** | **3.51** |
| *eutB* | ethanolamine ammonia-lyase subunit alpha | **4.46** | **4.00** |
| *eutC* | ethanolamine ammonia-lyase subunit beta | **3.67** | **2.90** |
| *eutK* | ethanolamine utilization microcompartment protein EutK | **4.38** | **3.02** |
| *eutL* | ethanolamine utilization microcompartment protein EutL | **4.06** | **2.90** |
| **Uptake and degradation of polyamines** | | | |
| *potB* | spermidine/putrescine ABC transporter permease PotB | **n.d.e.** | **2.16** |
| *potC* | spermidine/putrescine ABC transporter permease PotC | **n.d.e.** | **2.99** |
| *potD* | spermidine/putrescine ABC transporter substrate-binding protein PotD | **2.35** | **3.29** |
| *potF* | spermidine/putrescine ABC transporter substrate-binding protein PotF | **2.69** | **2.99** |
| *potG* | putrescine ABC transporter ATP-binding subunit PotG | **3.13** | **2.07** |
| *potH* | putrescine ABC transporter permease PotH | **3.09** | **2.44** |
| *potI* | putrescine ABC transporter permease PotI | **3.66** | **3.09** |
| *puuE* | 4-aminobutyrate transaminase | **2.87** | **n.d.e.** |
| *patA* | putrescine aminotransferase | **n.d.e.** | **2.77** |
| *gabT* | 4-aminobutyrate--2-oxoglutarate transaminase | **6.83** | **2.03** |

*n.d.e = not differentially expressed


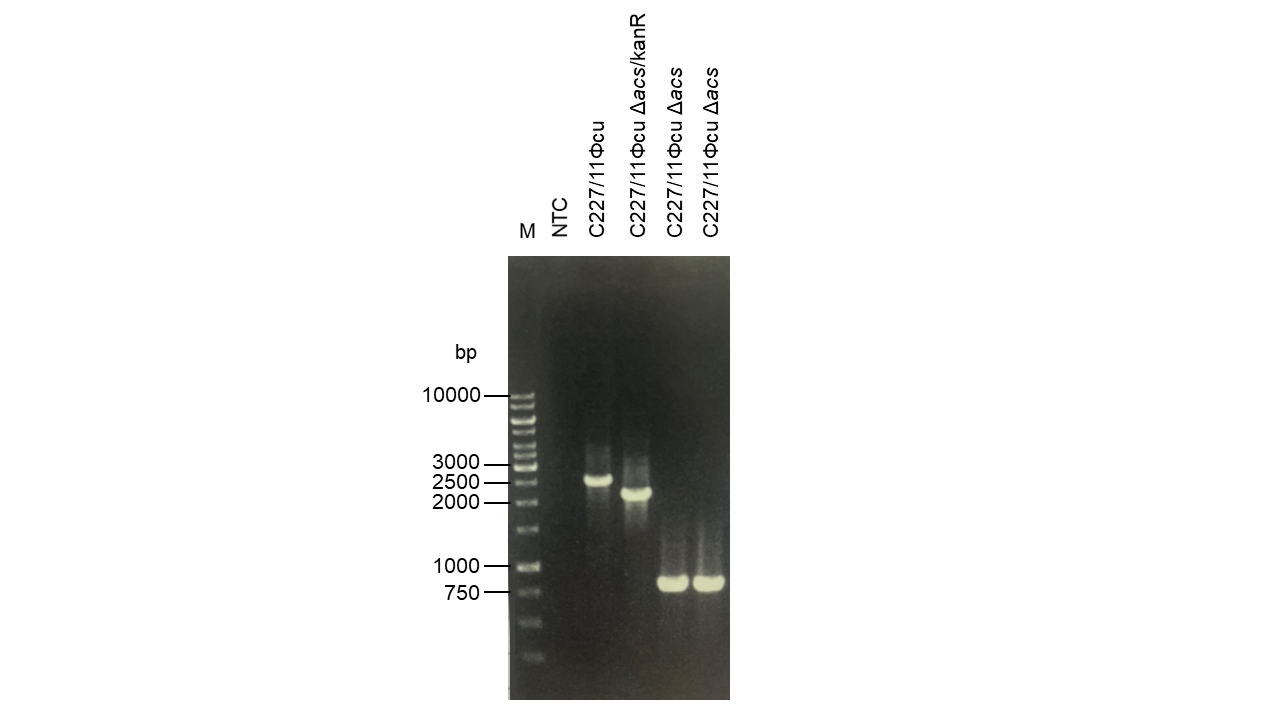


**Figure S1:** Agarose gel electrophoresis to confirm the incorporation and subsequent deletion of kanamycin resistance cassette to construct isogenic *acs* deletion mutant of C227/11Φcu.

The sample with DNA from C227/11Φcu as template shows an amplicon with a size of ~2500bp. The sample with DNA from C227/11Φcu Δ*acs*/kanR shows a band at ~2100 bp which indicates the incorporation of the kanamycin resistance cassette (kanR). The removal of kanR was confirmed for two samples with bands at ~750 bp. The mutant strain C227/11Φcu Δ*acs* was successfully prepared. Marker (1 kb DNA ladder, GeneRuler, Thermo Scientific, USA) and no template control (NTC) in PCR are additionally shown.
